# An *in vitro* assay for enzymatic studies on human ALG13/14 heterodimeric UDP-*N*-acetylglucosamine transferase

**DOI:** 10.3389/fcell.2022.1008078

**Published:** 2022-09-19

**Authors:** Chun-Di Wang, Si Xu, Shuai Chen, Zheng-Hui Chen, Neta Dean, Ning Wang, Xiao-Dong Gao

**Affiliations:** ^1^ Key Laboratory of Carbohydrate Chemistry and Biotechnology, Ministry of Education, School of Biotechnology, Jiangnan University, Wuxi, China; ^2^ Department of Biochemistry and Cell Biology, Stony Brook University, New York City, NY, United States; ^3^ State Key Laboratory of Biochemical Engineering, Institute of Process Engineering, Chinese Academy of Sciences, Beijing, China

**Keywords:** N-glycosylation, lipid-linked oligosaccharide (LLO), ALG glycosyltransferases, ALG13/14 UDP-N-acetylglucosamine transferase, ALG13 isoforms, congenital disorders of glycosylation (CDG)

## Abstract

The second step of eukaryotic lipid-linked oligosaccharide (LLO) biosynthesis is catalyzed by the conserved ALG13/ALG14 heterodimeric UDP-*N*-acetylglucosamine transferase (GnTase). In humans, mutations in ALG13 or ALG14 lead to severe neurological disorders with a multisystem phenotype, known as ALG13/14-CDG (congenital disorders of glycosylation). How these mutations relate to disease is unknown because to date, a reliable GnTase assay for studying the ALG13/14 complex is lacking. Here we describe the development of a liquid chromatography/mass spectrometry-based quantitative GnTase assay using chemically synthesized GlcNAc-pyrophosphate-dolichol as the acceptor and purified human ALG13/14 dimeric enzyme. This assay enabled us to demonstrate that in contrast to the literature, only the shorter human ALG13 isoform 2, but not the longer isoform 1 forms a functional complex with ALG14 that participates in LLO synthesis. The longer ALG13 isoform 1 does not form a complex with ALG14 and therefore lacks GnTase activity. Importantly, we further established a quantitative assay for GnTase activities of ALG13- and ALG14-CDG variant alleles, demonstrating that GnTase deficiency is the cause of ALG13/14-CDG phenotypes.

## Introduction

Asparagine (N)-linked glycosylation is a posttranslational modification that directly affects the structure and function of glycoproteins, thus influencing a multitude of essential biological processes (Takahashi et al., 2016; Loke et al., 2016; Dwyer and Esko, 2016; Esmail and Manolson, 2021). Protein N-glycosylation begins with the assembly of a lipid-linked oligosaccharide (LLO) precursor whose synthesis starts on the cytosolic side of the endoplasmic reticulum (ER) membrane and finishes in its lumen (Supplementary Figure S1) (Aebi, 2013). The sequential addition of sugars to dolichol pyrophosphate (PDol) at the ER membrane by the ALG (asparagine-linked glycosylation) glycosyltransferases (GTases) produce a LLO containing fourteen sugars, including two GlcNAcs (Gn), nine mannoses (M) and three glucoses (G) (Li et al., 2019). Once assembled, the G3M9Gn2 oligosaccharide is transferred from the lipid to nascent proteins by oligosaccharyltransferase (Cherepanova et al., 2016; Shrimal and Gilmore, 2019). In humans, defects in LLO synthesis or transfer to proteins cause inherited metabolic diseases known as congenital disorders of glycosylation (CDG) that manifest as a broad range of multisystem disorders or even embryonic lethality (Jaeken, 2020; Pascoal et al., 2020). Based on recent statistical data, the number of identified ALG-CDGs is rapidly increasing, underscoring the significance of LLO synthesis to human disease (Sosicka et al., 2021).

Fourteen *ALG* genes are required for *N-*glycosylation in the ER (Schwarz and Aebi, 2011; Aebi, 2013). Among them, the *ALG13* and *ALG14* are unique in that the enzymes they encode interact to form a heterodimeric UDP-N-acetylglucosamine transferase (GnTase). Unlike other ALG GTases which may function alone, ALG13/14 GnTase only possesses activity when they interact with each other (Gao et al., 2004; Noffz et al., 2009). This dimeric ALG13/14 catalyzes the second step of LLO synthesis, transferring a β1,4-linked *N*-acetylglucosamine (GlcNAc) from UDP-GlcNAc to GlcNAc-pyrophosphate-dolichol (Gn-PDol) (Supplementary Figure S2) (Bickel et al., 2005; Chantret et al., 2005; Gao et al., 2005). ALG13/14 GnTase is widely conserved in eukaryotes. Most of our knowledge about the function of ALG13/14 GnTase comes from biochemical and genetic analyses in *S. cerevisiae*. Yeast Alg13 (yAlg13) is a small, soluble cytosolic protein that contains the GnTase catalytic subunit, while Alg14 (yAlg14) is a membrane protein that recruits soluble yAlg13 to the cytosolic face of the ER. Both yAlg13 and yAlg14 are essential for viability (Bickel et al., 2005; Chantret et al., 2005; Gao et al., 2005) and yAlg13 is inactive unless bound to yAlg14 (Bickel et al., 2005; Chantret et al., 2005). Because of its complex structure, in which the active heterodimer consists of both a hydrophilic and hydrophobic subunit, the development of an *in vitro* quantitative assay for studying the enzymatic properties of ALG13/14 GnTase has been hindered for a long time. Recently, liquid chromatography/mass spectrometry (LC–MS)-based techniques have been used for developing *in vitro* quantitative assays to study the enzymatic activity of ALG GTases (Li et al., 2017a; Li et al., 2017b; Wang et al., 2022; Xiang et al., 2022), but as yet, these have not been tested for their suitability for ALG13/14.

Phylogenetic analysis of ALG14-ALG13 homologs revealed their ancient origin in eukaryotes (Meyer et al., 2022). While ALG14 is structurally similar across eukaryotes, ALG13 has evolved differently: fungal and plant cells only possess a short form of the ALG13 protein, while vertebrates have multiple isoforms. In humans, four ALG13 isoforms generated by alternative splicing have been described (https://www.uniprot.org-ALG13, identifier: Q9NP73). Among them, isoform 1 (ALG13-iso1) and 2 (ALG13-iso2) are found in all mammals. ALG13-iso1 is the longest isoform of ALG13, consisting of 1,137 amino acids, while ALG13-iso2 is shorter, consisting of 165 amino acids. This short ALG13-iso2 shows more than 30% sequence similarity with animal, fungi and plant (Supplementary Figure S3). Co-expression of human ALG13-iso2 and ALG14 complemented the growth defect of yeast *alg13* and *alg14* mutant cells, indicating ALG13-iso2 is catalytically active when complexed with ALG14 (Gao et al., 2005). Studies have shown that it is possible to completely delete the ALG13-iso1 in a mouse model, but not the isoform 2 (Gao et al., 2019), suggesting the ALG13-iso2 is essential for glycosylation. On the other hand, ALG13-iso1 knockout mice have an increased susceptibility to epileptic seizures (Gao et al., 2019; Huo et al., 2020). Thus the biological function of ALG13-iso1 as it relates to glycosylation remains undefined.

Pathogenic mutations in human *ALG13* or *ALG14* cause severe neurological disorders with a multisystem phenotype. ALG13 variants underlie infantile-onset developmental and epileptic encephalopathy (DEE) (Timal et al., 2012; Ng et al., 2020; Alsharhan et al., 2021; Datta et al., 2021), while *ALG14* gene mutants present as early lethal neurodegeneration with myasthenic and myopathic features, also known as congenital myasthenic syndrome (CMS) (Judith et al., 2013; Schorling et al., 2017; Datta et al., 2021; Katata et al., 2022). Given the essential role of ALG13/14 GnTase in ER N-glycosylation, it has been assumed that these phenotypes fall under the umbrella of CDG. Surprisingly, however, unlike other ALG-CDGs, most ALG13- and ALG14-CDG variants show a normal glycosylation pattern on transferrin, the commonly used biomarker for CDG (Schorling et al., 2017; Alsharhan et al., 2021). Thus direct evidence linking ALG13/14 GnTase activity to the DEE or CMS phenotypes remains elusive.

In this work, we co-expressed human ALG13 and ALG14 in *E. coli* and purified recombinant ALG13/14 complex from a membrane fraction. Using chemically synthesized Gn-PDol as acceptor substrate, we confirmed the GnTase activity of recombinant ALG13/14. We used this purified enzyme to study the kinetic properties of ALG13/14 GnTase with an *in vitro* quantitative assay utilizing LC–MS. Furthermore, this *in vitro* assay system was used to compare the GnTase activities of the different human ALG13 isoforms. Our results confirmed that ALG13-iso2, but not ALG13-iso1, forms a dimeric complex with ALG14 on the cytosolic face of the ER membrane and contributes to GnTase activity for LLO synthesis. We also applied this assay to demonstrate the GnTase activities of ALG13- and ALG14-CDG variants. These variants display severe enzymatic defects *in vitro*, thus providing evidence for a genetic link between GnTase activity and ALG13-related DEE or ALG14-related CMS.

## Material and methods

### Plasmids, strains, cell lines and culture conditions

The plasmids used in this study and their important features are described in Table 1. Standard molecular biological techniques were used for plasmid construction, and mutations that altered various amino acids (see Table 2) were introduced using overlapping PCR with mutagenic primers (BGI, Shenzhen, China) and verified by DNA sequence analysis.

**TABLE 1:** Plasmids used in this study.

Plasmid	Description
pET26b-pelB-*FLAG*-*ALG13*	Kanamycin, FLAG-ALG13 expressed in *E.coli*
pCDFDuet-*6His-ALG14*	Streptomycin, 6His-ALG14 expressed in *E.coli*
pME	Mammalian cell expression vector
pME-*3HA-ALG13 iso1*	3HA-ALG13 iso1 expressed in pME
pME-*3HA-ALG13 iso2*	3HA-ALG13 iso2 expressed in pME
pME-*ALG14-3FLAG*	ALG14-3FLAG expressed in pME
YEp352GAPII	URA3/2μ yeast shuttle vector containing TDH3 promoter
YEp352GAPII-*3HA-ALG13 iso1*	3HA-ALG13 iso1 expressed in YEp352GAPII
YEp352GAPII-*3HA-ALG13 iso2*	3HA-ALG13 iso2 expressed in YEp352GAPII
YEp351GAPII	LEU2/2μ yeast shuttle vector containing TDH3 promoter
YEp351GAPII-*ALG14-FLAG*	ALG14-3FLAG expressed in YEp351GAPII

**TABLE 2:** Relative activity of ALG13 and ALG14 variants.

Gene	cDNA position	Protein position	Sex	Age	Transferrin	Relative activity (%)
ALG13	c.50T>A	p.I17N	Female	NM	ND	11
	c.207–209 delAGA	p.E69del	Female	NM	ND	18
	c.241G>A	p.A81T	Female	11 years old	ND	27
	c.280A>G	p.K94E	Male	Died in 1 years old	Abnormal	9
	c.320A>G	p.N107S	Female & Male	From 4 months to 7 years old	Near normal	8
	C.421-422AC>TT	p.T141L	Male	59 years old	ND	26
ALG14	c.194C>T	p.P65L	Female	62 years old	Near normal	80
	c.310C>T	p.R104X				23
	c.220G>A	p.D74N	Female & Male	Died in 8 months	Normal	22
	c.220G>A	p.D74N	Male	Died in 4 months	ND	22
	c.326G>A	p.R109Q				26
	c.220G>A	p.D74N	Female	Died in 6.5 months	ND	22
	c.422T>G	p.V141G				28

Relative activity was defined as the conversion rate of the mutants/the conversion rate of the wild type under standard assay conditions. The relative activity was the average of the three results. NM: not mention; ND: not detect.

Human embryonic kidney cells, HEK293, were cultured in DMEM supplemented with 10% FBS (FCS, Biological Industries, Beit Haemek, Israel), penicillin and streptomycin (1 μg/ml) in a humidified 5% CO_2_ atmosphere at 37°C.

### Expression and extraction of recombinant ALG13 and ALG14 proteins in *E. coli*

*E. coli* Rosetta cells (DE3, Merck, NJ, United States) harboring both pET26b-pelB-*FLAG-ALG13-iso2* and pCDFDuet-*6His-ALG14* plasmids were cultured in 200 ml of Terrific-Broth medium (TB, 1.2% tryptone, 2.4% yeast extract and 0.5% glycerol) at 37°C to an OD600 of 1.0 and then cooled to 16°C. After adding 0.1 mM isopropyl-β-d-thiogalactopyranoside (IPTG, Sangon Biotech, Shanghai, China), the cells were incubated for additional 18 h–24 h at 16°C to induce protein expression. The harvested cells were resuspended in 15 ml lysis buffer (150 mM NaCl, 50 mM Tris/HCl, pH 8.0) and homogenized by sonication. The cell lysate was centrifuged at 4,000×*g* to remove debris, and the supernatant was collected (this supernatant was used as the “crude extract”). To obtain the membrane fraction containing ALG13/14 complex, the crude extract was further centrifuged at 12,000×*g* to collect precipitated membranes. Any remaining cytosolic ALG13 was removed by washing the membranes with 15 ml lysis buffer three times. This washed membrane fraction was then solubilized by resuspension in 5 ml lysis buffer containing 1% Triton X-100 and incubated on ice for 15 min to obtain the detergent extract. This detergent extract was used for purification of the recombinant ALG13/14 complex following the procedure described below. This detergent extract was also used for the co-immunoprecipitation experiment presented in Figure 2A.

### Purification of the ALG13/14 complex from *E. coli* membrane lysates

The detergent extract prepared from the *E. coli* cells co-expressing FLAG-ALG13-iso2 and His-ALG14 was used to purify the recombinant ALG13/14 complex. First, His-tagged ALG14 proteins, including the recombinant FLAG-ALG13-iso2/His-ALG14 complex in detergent extract, were trapped by a HisTrap HP affinity column (GE Healthcare Life Sciences). Their eluted solution was then incubated with anti-FLAG Affinity Matrix (Sigma–Aldrich, MO, United States) to enrich the ALG13/14 complex and remove the free His-ALG14. After elution with FLAG peptide, the purified ALG13/14 complex was analyzed by native PAGE and SDS–PAGE followed by silver staining to confirm the purity. Native PAGE was performed using a Blue/Clear Native PAGE Electrophoresis Kit (Real-time, Beijing, China) according to the manufacturer’s instructions.

### Preparation of protein extracts and membrane fractions from HEK293 cells

HEK293 cells were seeded in 6-well plate and transfection was performed when the cell density was 80%–90%. Transfection was performed using Lipofectamine 2,000 (Thermo Fisher Scientific, MA, United States) according to the manufacturer’s instructions. 4 μg plasmid (when co-transfect two plasmids, 2 μg per plasmid) and 5 μL lipofectamine 2000 were diluted using 250 μl OPTI-MEM respectively, after incubated at room temperature for 5 min, then mixed and incubated for another 20 min. The mixed reagent was added in HEK293 cells which grown in 2 ml DMEM medium with 10% FBS. Changed the mixture to DMEM medium with 10% FBS after 8 h and harvested the cell after 72 h.

**FIGURE 1:**
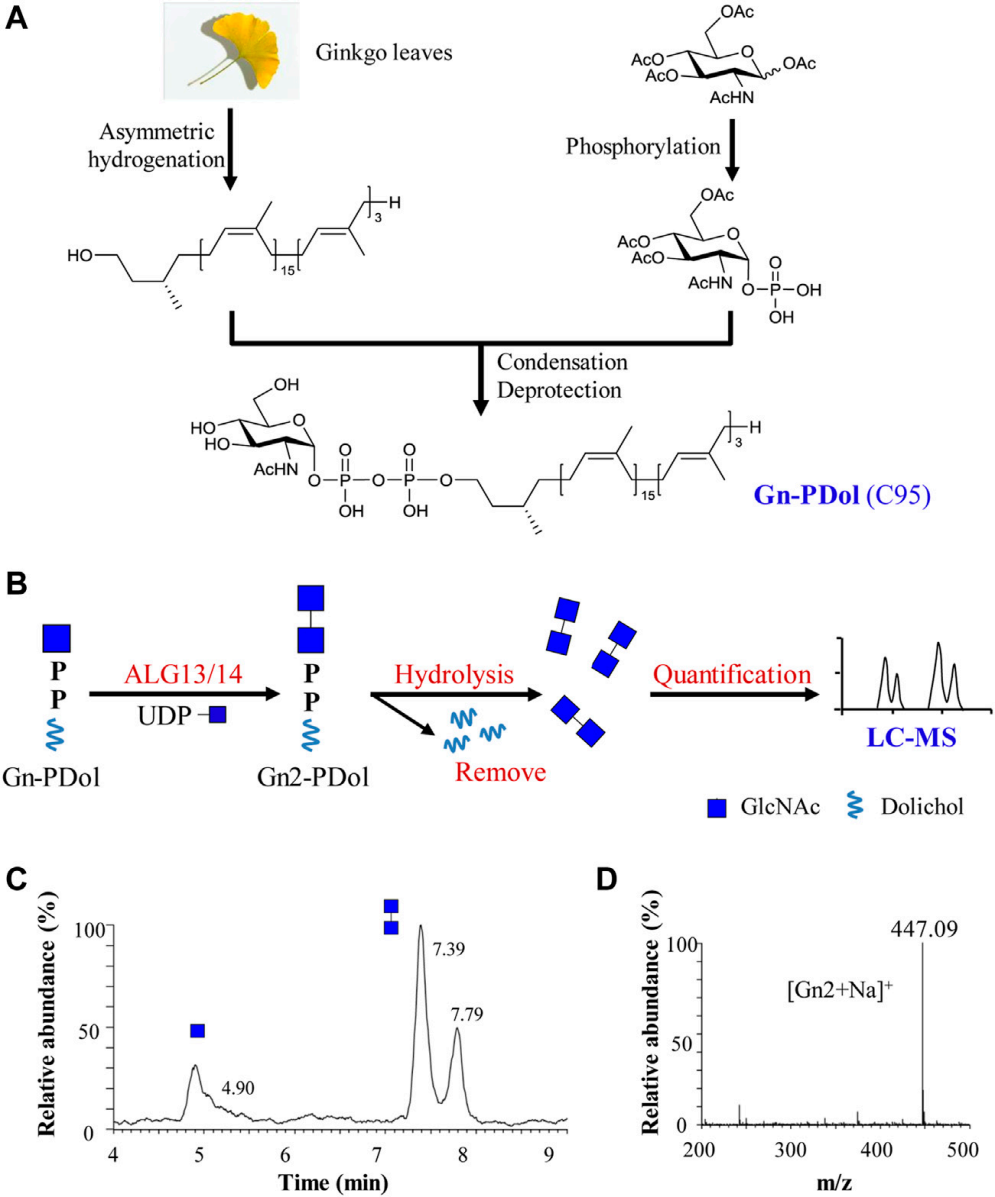
Preparation and feasibility test of lipid-linked acceptor GlcNAc-PP-Dol (Gn-PDol, C95). **(A)** The semisynthetic strategy of lipid-linked acceptor Gn-PDol (C95); **(B)** Work flow of an in vitro quantitative GnTase assay for ALG13/14 complex; **(C)** The UPLC chromatogram of glycans released from ALG13/14 reaction mixture. Reaction was performed using HEK293 cells membrane fraction with the standard reaction mixture and incubated for 1 h. Peaks eluted earlier represent Gn and eluted later represent Gn2; **(D)** ESI-MS spectra of peaks eluted at 7.39 and 7.79 min in UPLC correspond to Gn2 ([Gn2+Na]+).

To prepare the protein extract, 1 × 10^6^ cells were solubilized in HEPES buffer with 1% NP-40 (25 mM HEPES, pH 7.4, 150 mM NaCl, 1%, 1 mM PMSF and 1 × Protein inhibitor cocktail) and incubated on ice for 30 min. Cell lysates were centrifuged at 15,000×*g* for 10 min at 4°C. The resulting supernatants were collected as the protein extracts. To prepare the membrane fraction which contain endogenous ALG13/14, HEK293 cells (1 × 10^6^ cells) were resuspended in 1 ml of hypotonic buffer (10 mM Tris/HCl, pH 7.4, 1.5 mM MgCl_2_, 10 mM KCl, protease inhibitor), followed by incubation on ice for 30 min. Cells were then homogenized by passing them 36 times through a homogenizer. Nuclei and cell debris were pelleted by centrifugation at 2,500×*g* for 5 min at 4°C. The supernatant was collected and centrifuged again at 12,000×*g* for 30 min at 4°C. The obtained membrane fraction (equivalent to 30 μg membrane protein) was used as the enzyme fraction of ALG13/14 GnTase in studies that characterized the suitability of synthetic Gn-PDol (C95) as substrate, as presented in Figure 1C.

### Western blotting and co-immunoprecipitation

*E. coli* co-expressing FLAG-ALG13-iso2 and 6His-ALG14 were cultured, and crude extracts were prepared as described above. Ten micrograms of this crude extract was separated by 12% SDS–PAGE analysis and transferred to a PVDF membrane (Bio-Rad, CA, United States). Immunoblotting was performed with an anti-His mouse antibody (TransGen Biotech, Beijing, China) and anti-FLAG mouse monoclonal antibody (TransGen Biotech, Beijing, China) as the primary antibody and the anti-mouse IgG-horseradish peroxidase (HRP) conjugate (TransGen Biotech, Beijing, China) as the secondary antibody followed by chemiluminescence (ECL) detection (Bio-Rad, CA, United States). For co-immunoprecipitation, 6His-ALG14 or FLAG-ALG13-iso2 was immunoprecipitated with Ni-NTA His binding resin (TransGen Biotech, Beijing, China) or anti-FLAG Affinity Matrix (Sigma–Aldrich, St. Louis, MO, United States) as described elsewhere (Gao et al., 2005). After separation by 12% SDS–PAGE, the precipitated proteins were transferred to an Immobilon-PVDF membrane. The membrane was incubated with an anti-FLAG mouse monoclonal antibody or anti-His mouse monoclonal antibody, washed, and incubated with anti-mouse IgG–HRP conjugate. Immunoreactive bands were visualized by ECL.

Similarly, HEK293 cells co-expressing HA-ALG13-iso1/FLAG-ALG14 or HA-ALG13-iso2/FLAG-ALG14 were cultured, and their protein extracts were prepared as described above. Ten μg of each protein extract was analyzed using western blot. Immunoblotting was performed with anti-FLAG mouse monoclonal antibody (Sigma-Aldrich, MO, United States) and anti-HA rabbit polyclonal antibody (Cell Signaling Technology, MA, United States) as the primary antibody and the anti-mouse IgG-HRP conjugate or the anti-rabbit IgG-HRP conjugate (TransGen Biotech, Beijing, China) as the secondary antibody. Immunoreactive bands were visualized by ECL. For co-immunoprecipitation assays, protein extracts were immunoprecipitated with anti-HA rabbit polyclonal antibody followed by incubation with 40 μl Protein A + G Agarose (Beyotime Shanghai, China) for 3 h to pull down the HA-tagged ALG13-iso1 or ALG13-iso2 protein. After washing 3 times with HEPES buffer, the immunoprecipitate was solubilized, separated by SDS-PAGE and analyzed by western blotting with anti-FLAG mouse monoclonal antibody to detect the co-immunoprecipitated FLAG-ALG14.

### Purification of the ALG13-iso from HEK293 cells

For the purification of ALG13-iso1 and ALG13-iso2, HEK293 cells (3 × 10^6^ cells) expressing HA-ALG13-iso1 or HA-ALG13-iso2 were harvested and the protein extract was prepared as described above. The protein extract was incubated with anti-HA rabbit polyclonal antibody and 40 μl Protein A + G Agarose for 4 h to enrich the HA-tagged ALG13-iso1 or ALG13-iso2 protein. After washing the agarose by PBS for 3 times, the agarose-bound immunoprecipitated proteins were divided into two parts. One part was immunoblotted with anti-ALG13 rabbit polyclonal antibody (Proteintech, Wuhan, China) to detect HA-ALG13 while the other part was employed to test the GnTase activity.

### Quantitative assay of ALG13/14 activity

The standard reaction mixture for ALG13/14 GnTase assay contained in a volume of 50 μl: 28 mM Tris/HCl, pH 7.5, 0.3% NP-40, 23% glycerol, 40 μM Gn-PDol, 2 mM UDP-GlcNAc (Sigma-Aldrich, MO, United States) and protein. After incubation at 37°C, the reaction was stopped by heating to 100°C. The specific amount of protein included and reaction times are described in detail in each section. The donor concentration using 2 mM, which was 50 folds than the substrate Gn-PDol concentration. ALG13/14 can only transfer one GlcNAc from UDP-GlcNAc to Gn-PDol, so the donor concentration was excess which could facilitate the reaction.

After terminating the reaction, glycans were released by adding 50 μl of HCl (40 mM) to the reaction and shifting the temperature to 100°C for 1 h. The hydrolyzed glycans were purified as described (Li et al., 2017a; Li et al., 2017b; Xu et al., 2018; Li et al., 2019; Xiang et al., 2022). The desalted sample was injected into a Dionex Ultimate 3,000 UPLC (Thermo Scientific, MA, United States) and separated using a Waters Acquity UPLC BEH Amide Column (1.7 μm 2.1 × 150 mm) at a flow rate of 0.2 ml/min. The UPLC conditions were same as previous report while the ESI-MS range was adjusted to 100–1,500 m/z (positive mode). The oligosaccharide transfer rate was calculated using the peak intensity in LC-ESI-MS through Xcalibur (Version 2.0, Thermo Scientific, MA, United States).

### Characterization of recombinant ALG13/14 GnTase

Enzymatic assays were performed using purified protein with the final concentration as 0.3 ng/μl (Figures 3B–E) or 0.2 ng/μl (Figure 3F) under the standard conditions for 15 min as described above. The optimized reaction conditions were as follows: temperature from 10°C to 60°C, pH from 4.0 mM to 10.0 mM, 10 mM ions (Co^2+^, Mn^2+^ and Mg^2+^, respectively) or 10 mM EDTA for depletion conditions. The apparent *K*_*m*_ and *k*_*cat*_ values for Gn-PDol (20 μM–100 μM) were calculated by nonlinear regression curve fitting (GraphPad Prism 8.0, GraphPad Software Inc.). The donor specificity of ALG13/14 was confirmed using different donor (UDP-Glc, UDP-GlcNAc and GDP-Man) with same concentration (2 mM).

The stereochemistry of the linkage between two GlcNAc residues in Gn2-PDol (C95), the product of ALG13/14 GnTase, was confirmed by using yeast Alg1 (yAlg1) MTase. Purified recombinant His-yAlg1ΔTM and GDP-Man were added to the recombinant ALG13/14 reaction system, followed by 1 h of incubation at 30°C. The products were analyzed by LC–MS as described above.

### Complementation assay of ALG13-iso/14 in *S. cerevisiae*

The yeast strain XGY186 (as in W303a; *∆alg13::His* ^*+5*^ *; ∆alg14::Kan* ^*+*^ and *GAL1/10pr-3HA-yALG14*/*yALG13-FLAG::trp1*), contains a deletion of *ALG13* and *ALG14* in the chromosome and a *GAL1/10* promoter-driven *yALG13* and *yALG14*. This strain was used to test for the ability of human ALG13 different isoforms and ALG14 to complement growth in the presence of glucose. The *URA3-*marked YEp352GAPII-*3HA-ALG13-iso1* or YEp352GAPII-*3HA-ALG13-iso2* plasmid was co-transformed with the *LEU2*-marked YEp351GAPII-*ALG14-FLAG* into XGY186 and selected on SG (-Ura-Leu) plates. Transformants were cultured in medium containing glucose, followed by growth on solid YPA medium (1% yeast extract, 2% peptone, 50 mg/L adenine) supplemented with 2% glucose (YPAD) or 2% galactose (YPAG) at 30°C for 2 days.

### Statistics and reproducibility

The enzymatic properties, kinetic analysis and CDG variants relative activity experiments were independently repeated three times. Sample size, mean, and kinetic standard deviation (SD) value are provided in the figure, figure legend and table footnote. Kinetic parameters were determined by nonlinear regression fitted to Michaelis-Menten equation in GraphPad Prism 8.0.

## Results

### Preparation of the acceptor substrate for ALG13/14 UDP-GlcNAc transferase

The human ALG13/14 (ALG13/14) complex catalyzes the addition of a β1,4-linked GlcNAc to Gn-PDol to produce Gn2-PDol in the DLO synthetic pathway. We aimed to establish an *in vitro* assay system for quantitatively detecting UDP-GlcNAc transferase (GnTase) activity. We previously described an LC–MS-based quantitative assay for LLO mannosyltransferases (Li et al., 2019) and this assay was modified for quantitative measurements of ALG13/14 GnTase activity with a suitable Gn-PDol acceptor substrate. Unlike other ALG GTases, which can use phytanyl-linked oligosaccharides as their acceptor substrates for enzymatic studies (Li et al., 2017a; Li et al., 2017b; Xu et al., 2018; Li et al., 2019; Xiang et al., 2022), ALG13/14 GnTase does not recognize GlcNAc-pyrophosphate-phytanyl as the acceptor (data not shown). Therefore, we tried to prepare its natural Gn-PDol acceptor using semisynthetic methods. Since the lipid tails of the DLO in mammalian cells usually contain 17–21 isoprene units (C85–C105), we synthesized a Gn-PDol (C95) substrate that contains 19 isoprene units of lipid tails (Figure 1A). A polyprenol (C95) obtained from ginkgo leaves was applied to the asymmetric hydrogenation to generate dolichol, which was coupled to phosphorylated GlcNAc to obtain the target substrate Gn-PDol (C95, Figure 1A). The product structure was characterized by mass and nuclear magnetic resonance (NMR) analyses. The detailed synthetic procedures and data will be published elsewhere.

To test if this Gn-PDol (C95) is a suitable substrate for ALG13/14 GnTase, the HEK293 cells membrane fraction (equivalent to 30 μg membrane protein, which was confirmed contain endogenous ALG13 and ALG14 using anti-ALG13 antibody and anti-ALG14 antibody, data not shown) was prepared and applied to a standard reaction mixture with a final volume of 50 μl, in which Gn-PDol (C95) and UDP-GlcNAc were used as the acceptor and donor substrates (see Material and methods for details). Hydrochloric acid was added to hydrolyze the saccharide moieties, followed by purification and detection using LC–MS for quantitative analysis (Figure 1B). Two group peaks appeared on UPLC (Figure 1C). The corresponding ESI-MS analysis showed that the first peak eluted at 4.90 min was [Gn + H]^+^, and the mass peak at m/z 222.12 (Supplementary Figure S4) was hydrolyzed from the substrate Gn-PDol. The second group peak eluted at 7.39 min and 7.79 min was [Gn2+Na]^+^, with the mass peak m/z: 447.09, which was hydrolyzed from the product Gn2-PDol (Figures 1C,D). The two peaks of Gn2 have been proven to be glycan anomeric isomers, which were designated alpha (α) and beta (β) (Li et al., 2017a). The conversion rate was quantified by calculating the peaks intensity of Gn2 (two peaks)/peak intensity of (Gn + Gn2), which was 79.8% in Figure 1C. The boiled membrane fraction was applied to the standard reaction mixture as negative control, only one peak representing Gn was detected (data not shown). These results demonstrated the production of Gn2-PDol, indicating that Gn-PDol (C95) is a suitable substrate for ALG13/14 GnTase.

### Recombinant ALG13/14 complex possesses UDP-GlcNAc transferase activity

**FIGURE 2:**
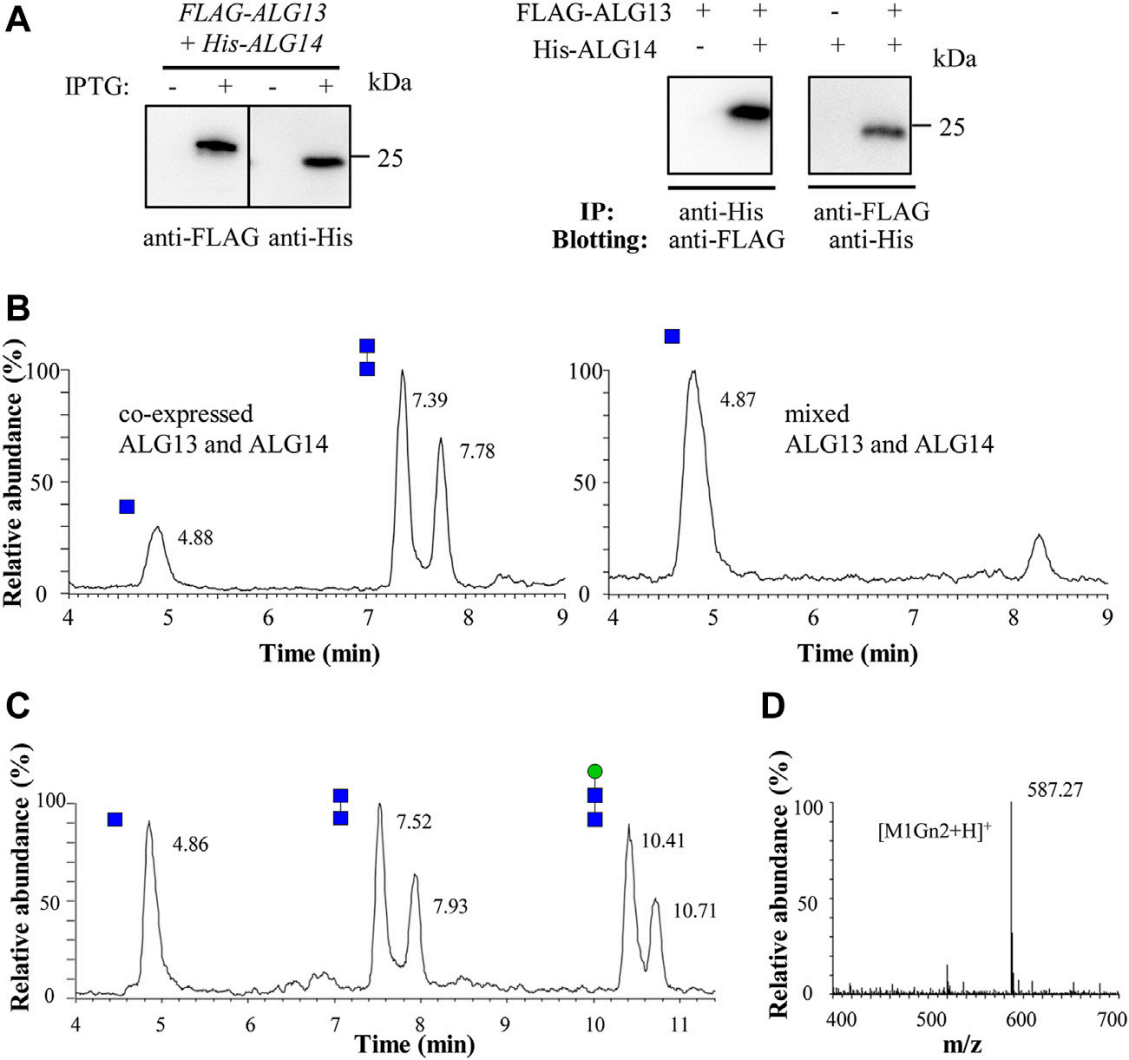
Recombinant ALG13/14 complex catalyzes the formation of Gn2-PDol. **(A)** Western blot and co-immunoprecipitation analysis of recombinant co-expressed ALG13 and ALG14 in *E. coli*. The crude extract prepared from *E. coli* cells was analyzed by western blot with anti-FLAG antibody or with anti-His antibody (left panel). The co-immunoprecipitation performed using Ni-NTA His binding resin or anti-FLAG Affinity Matrix, and immunoblotted with anti-FLAG antibody or anti-His antibody (right panel); **(B)** The UPLC chromatogram of glycans released from different reaction mixture. Reaction was performed using crude extract from co-expressed strain (5 μg total protein) or two different single expressed strain (5 μg total protein from each strain); **(C)** The UPLC chromatogram of glycans released from the recombinant ALG13/14 reaction mixture which add recombinant yAlg1 for additional 1 h. Peaks eluted at 10.41 min and 10.71 min represent Man-Gn2 (M1Gn2); **(D)** ESI-MS spectra of peaks eluted at 10.41 min and 10.71 min in UPLC correspond to M1Gn2 ([M1Gn2+H]^+^).

Although neither ALG13 nor ALG14 alone can rescue yeast *alg13* or *alg14* mutants, when co-expressed, human *ALG13* (*ALG13-iso2*) and *ALG14* can rescue the growth defect caused by either the loss of yeast *ALG13* or *ALG14* (Gao et al., 2005). These results demonstrated the formation of an active human ALG13/14 GnTase complex in yeast cells. Thus, to prepare active recombinant ALG13/14 complex, we chose to co-express human *ALG13-iso2* and *ALG14* in *E. coli*. Using N-FLAG- or His-tags, *ALG13-iso2* and *ALG14* were cloned into the pET26b and pCDFDuet expression vectors, respectively (Table 1). The resulting plasmids pET26b-pelB-*FLAG-ALG13-iso2* and pCDFDuet-*6His-ALG14* were transformed into *E. coli* Rosetta (DE3) cells. After induction with IPTG, the expression of both FLAG-ALG13-iso2 and His-ALG14 proteins was confirmed using anti-FLAG or anti-His antibodies (Figure 2A, left panel). Co-immunoprecipitation assays confirmed the formation of the ALG13/14 complex (Figure 2A, right panel). Immunoprecipitation of His-ALG14 with Ni-NTA His binding resin brought down the FLAG-ALG13-iso2 protein, which was detected by anti-FLAG antibody, and vice versa. These results suggested that ALG13/14 form a heterodimer in *E. coli*.

To determine if these recombinant proteins are active, GnTase activity of crude extracts was measured. Total protein (5 μg) prepared from *E. coli* that co-expressed ALG13-iso2 and ALG14 was incubated with the standard reaction mixture containing Gn-PDol (C95) and UDP-GlcNAc for 1 h, followed by detection of Gn2 product using LC–MS. The Gn2 product can be detected as two peaks at retention times of 7.39 min and 7.78 min (Figure 2B, left panel) with a mass of [Gn2+Na]^+^ (data not shown). When extracts prepared from two different *E. coli* strains (5 μg total protein from each strain), which individually expressed ALG13-iso2 and ALG14 were mixed and assayed for activity under the same conditions, no product peaks were detected (Figure 2B, right panel). These results indicate that the GnTase activity of the ALG13/14 complex cannot be obtained by mixing the individual recombinant ALG13 and ALG14 proteins. Instead, they imply that complex formation occurs *in vivo* and once formed, is stable.

To further confirm the stereochemistry of the GlcNAc-GlcNAc linkage in the newly formed product, we asked if it could be recognized as a substrate by yAlg1, the mannosyltransferase that catalyzes the reaction following ALG13/14 GnTase. yAlg1, adds β1,4-Man to GlcNAc(β1,4)GlcNAc-PDol (Gn2-PDol) with strict substrate specificity (Li et al., 2017a). To test this, recombinant yAlg1 was added to ALG13/14 GnTase reaction buffer with 2 mM GDP-mannose (GDP-Man) and incubated for another 1 h. After incubation, peaks corresponding to Man-Gn2 were observed at 10.41 min and 10.71 min, with a mass peak m/z of 587.27 (Figures 2C,D). The Man-Gn2 also was proved as glycan anomeric isomers. The ability of yAlg1 to produce this product implies the presence a β1,4 linkage in the GlcNAc-GlcNAc (Gn2) product of ALG13/14 GnTase. Taken together, our results demonstrated that co-expression of human *ALG13-iso2* and *ALG14* genes in *E. coli* can form an enzymatically active GnTase complex.

### Studying the enzymatic properties of the heteromeric ALG13/14 GnTase

**FIGURE 3:**
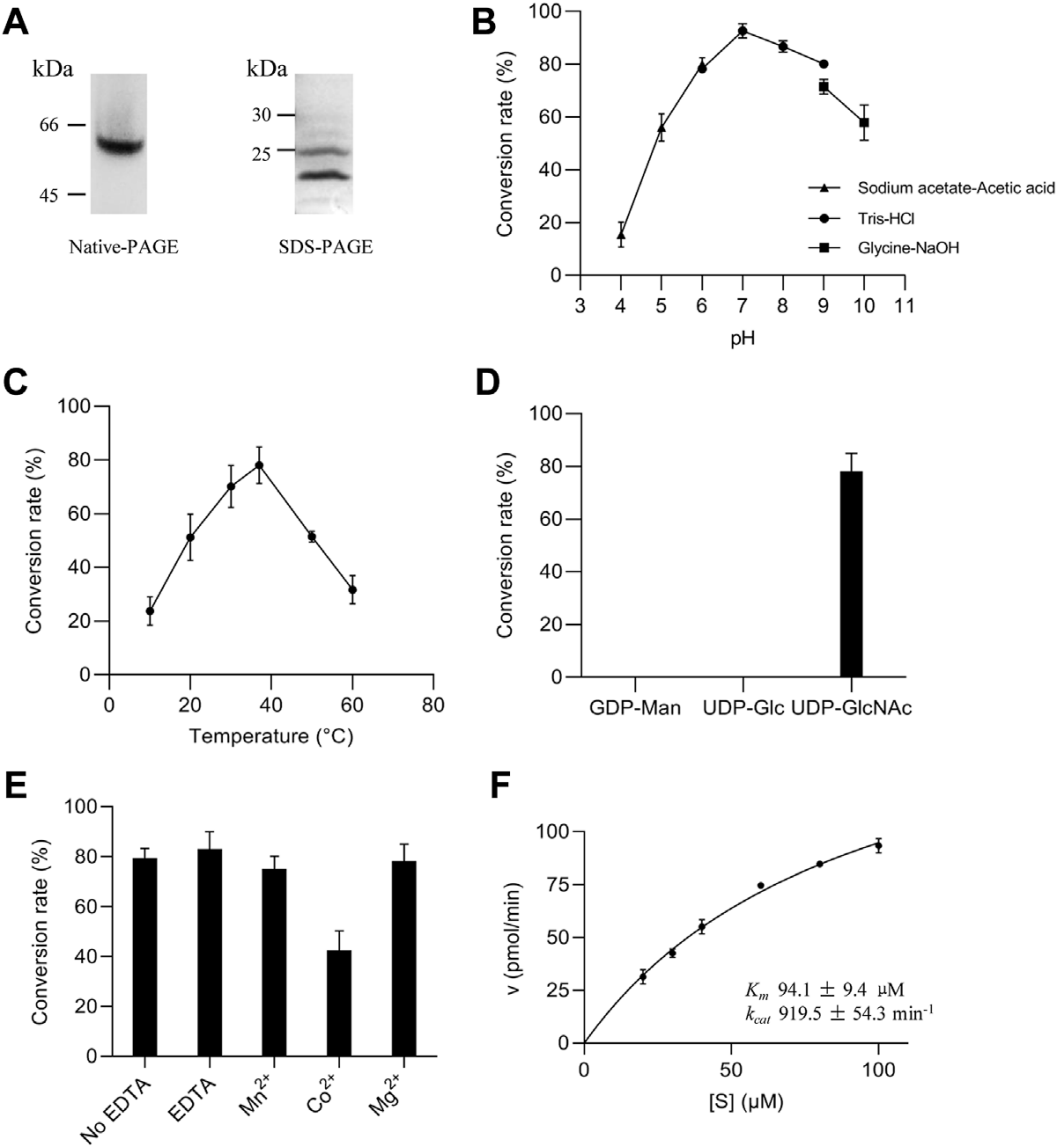
Enzymatic properties of ALG13/14 GnTase. **(A)** Native PAGE and SDS-PAGE analysis of purified heteromeric ALG13/14 complex; **(B)** Optimal pH was examined using various buffers, including sodium acetate-acetic acid buffer (pH 4.0, 5.0 and 6.0); Tris/HCl buffer (pH 6.0, 7.0, 8.0 and 9.0); glycine/NaOH buffer (pH 9.0 and 10.0); **(C)** Optimal temperature was evaluated at indicated temperatures (10°C, 20°C, 30°C, 37°C, 50°C and 60°C); **(D)** The specificity of the nucleotide sugar donor was studied by performing the reaction with 2 mM GDP-Man, UDP-Glc, or UDP-GlcNAc; **(E)** Divalent cation dependency was examined using 10 mM ions (Co^2+^, Mn^2+^ and Mg^2+^, respectively) or 10 mM EDTA for depletion conditions; **(F)** The *K*_*m*_ (94.1 μM ± 9.4 μM) and *k*_*cat*_ (919.5 min^−1^ ± 54.3 min^−1^) values for the substrate Gn-PDol whose concentration ranged from 20 μM to 100 μM, were calculated by nonlinear regression with a constant concentration of 2 mM UDP-GlcNAc. Each data point represents the mean ± SD value calculated from three independent experiments.

To further study its enzymatic properties, recombinant human ALG13/14 complex from *E. coli* harboring both pET26b-pelB-*FLAG-ALG13-iso2* and pCDFduet-*6His-ALG14* plasmids was purified as described in “*Material and methods*”. The purified protein was calculated as 2.6 mg/ml (0.1 ml), the concentration exhibiting a 500-fold compared with *E. coli* membrane extract. Native-PAGE analysis, in which the complex remains associated, revealed a single band with a molecular weight of approximately 45 kDa–66 kDa, while denaturing SDS–PAGE analysis revealed two bands, the individual ALG13-iso2 (25 kDa) and ALG14 (22 kDa) proteins (Figure 3A). Together, these results suggested that the purified recombinant ALG13/14 complex exists as a heterodimer.

Purified ALG13/14 was used to optimize reaction conditions (see Material and methods for details). As shown in Figures 3B–E, using 0.3 ng/μl recombinant proteins, our experiments demonstrated that the optimum pH and reaction temperature were 7.0°C and 37°C, respectively (Figures 3B,C). Moreover, of the three nucleotide sugars tested as donor substrates (UDP-GlcNAc, GDP-Man, and UDP-Glc), only UDP-GlcNAc was specifically recognized by the ALG13/14 complex (Figure 3D). Since additional divalent metal cations were required for most glycosyltransferase activities, the effects of various divalent metal ions on GnTase activity of ALG13/14 were tested. There were no improved activities confirmed in the presence of Mn^2+^, Mg^2+^ and EDTA, whereas Co^2+^ even inhibited the activity to some extent (Figure 3E). These results suggested that ALG13/14 GnTase does not require divalent metal ions for its enzyme activity. Furthermore, to investigate the kinetics of the recombinant ALG13/14, the rate of conversion to Gn2-PDol by purified recombinant ALG13/14 (0.2 ng/μl) was measured for the Gn-PDol (C95) acceptor from 20 mM to 100 mM with a fixed concentration of 2 mM UDP-GlcNAc as the donor substrate. The *K*_*m*_ value was calculated as 94.1 μM, and the *k*_*cat*_ value was 919.5 pmol/min by nonlinear regression fitted to Michaelis–Menten (Figure 3F).

### Assessing the GnTase activity of human ALG13 isoform 1

**FIGURE 4:**
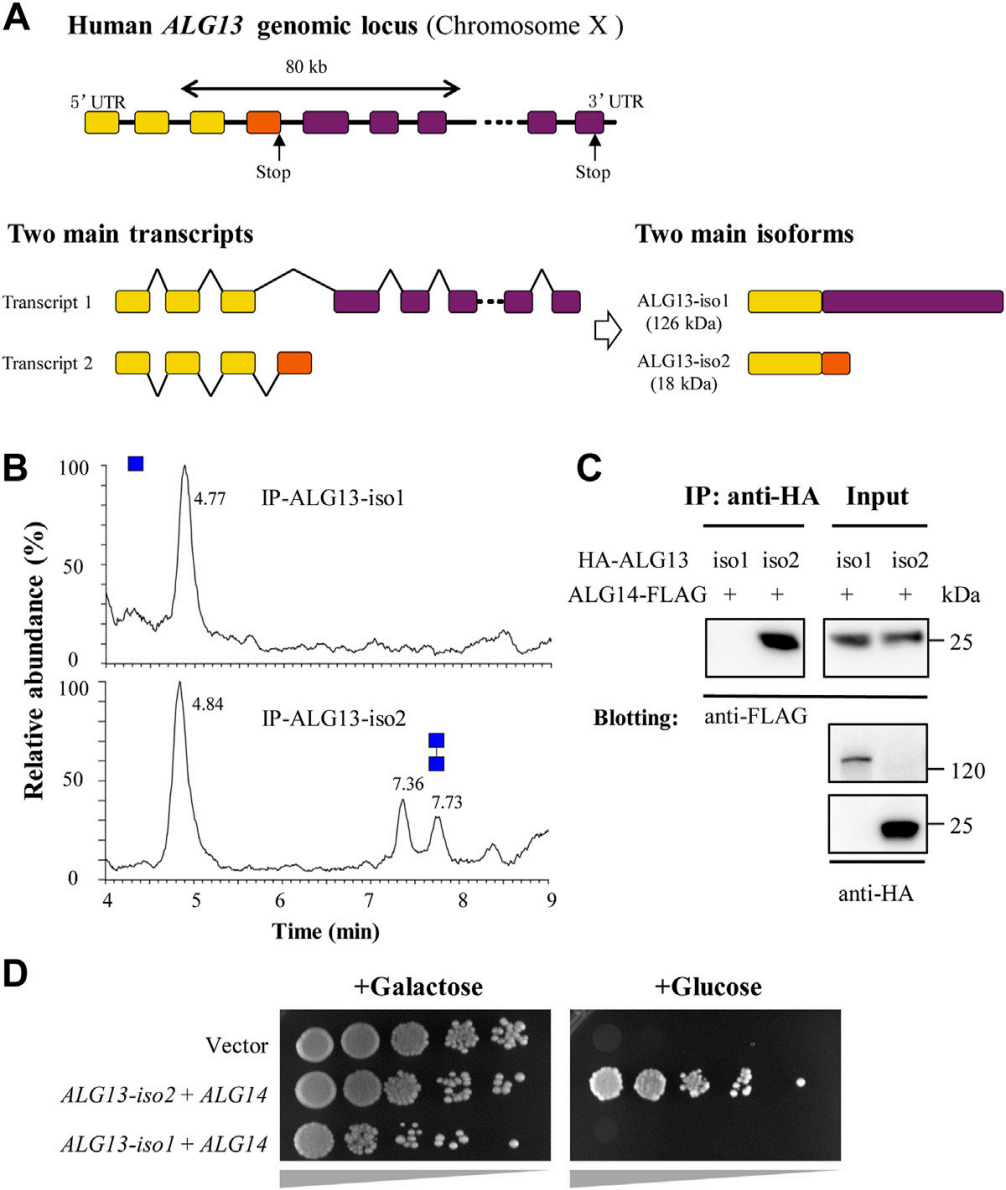
Human ALG13 isoform 1 fails to form GnTase complex with ALG14. **(A)** The gene map of ALG13-iso1 and ALG13-iso2. The two isoforms transcript from *ALG13* gene and share a same N-terminal while the C-terminal substantially different; **(B)** The UPLC chromatogram of glycans released from the reaction of ALG13 isoforms. HA-tagged ALG13 isoforms were expressed in HEK293 cell and enriched by using anti-HA agarose; **(C)** Co-immunoprecipitation analysis of ALG13 isoforms and ALG14. HA-tagged ALG13 isoforms and FLAG-tagged ALG14 were co-transfected in HEK293 cells, the co-immunoprecipitation performed using anti-HA agarose, and immunoblotted with anti-FLAG antibody; **(D)** Yeast complementary assay of ALG13 isoforms. An ALG13/14 deficient yeast strain XGY186 was grown under Gal promoter driving yAlg13 and yAlg14. ALG13 isoform and ALG14 were transfected and culture in YPA plate with galactose or glucose and incubated at 30°C for 2 days. iso: isoform.

As the canonical human isoform, the longer ALG13-iso1 contains not only an N-terminal glycosyltransferase 28 domain, but also several other domains including an ovarian tumor deubiquitinase domain (Ng et al., 2020). Compared to isoform 1, the short ALG13-iso2 lacks a large portion of the C-terminal region and uses an alternate 3′ terminal exon. The resulting iso2 protein shares N-terminal 127 amino acids with isoform 1, which contains the catalytic domain, and possesses a distinct C-terminal region consisting of 38 amino acids (Figure 4A). Among the ALG13 isoforms, only isoforms 1 and 2 contain the catalytic domain of ALG13/14 GnTase. It has been reported that ALG13-iso2 forms an active GnTase complex with ALG14 (Gao et al., 2005; Gao et al., 2008) (Figures 2, 3), but to date, no functional studies have ruled out that ALG13-iso1 also has catalytic activity.

To compare their GnTase activity, HA-tagged ALG13-iso1 protein was over-expressed alone in HEK293 and analyzed for enzymatic activity *in vitro* (Table 2). Cultured HEK293 cells (3 × 10^6^ cells) expressing HA-ALG13-iso1 were harvested and resuspended in 300 μl of HEPES buffer containing 1% Triton X-100 (see Material and methods for detailed composition). After incubation for 30 min on ice to solubilize membrane proteins, HA-ALG13-iso1 was immunoprecipitated from the cell lysate with anti-HA antibody and Protein A + G Agarose beads. The collected agarose beads were resuspended in 40 μL of reaction buffer. One half of this mixture (20 µl) was incubated with our standard reaction mixture to detect GnTase activity while the other half was subjected to SDS–PAGE analysis and immunoblotted with anti-ALG13 rabbit antibodies to confirm the expression and enrichment of HA-ALG13-iso1 proteins (data not shown). In parallel, HA-ALG13-iso2 was also expressed in HEK 293 cells and immunoprecipitated with anti-HA agarose (Table 2) (Gao et al., 2005). This pulled down ALG13-iso2 was selected as the positive control for GnTase activity because it can form an active GnTase complex with ALG14 in the ER membrane (Gao et al., 2005). Unexpectedly, by this assay HA-ALG13-iso1 did not show any GnTase activity, while HA-ALG13-iso2 could catalyze the formation of Gn2-PDol under the same reaction conditions (Figure 4B).

Because heterodimer formation is essential for ALG13/14 GnTase activity (Gao et al., 2008), we sought to determine the efficiency of complex formation between human ALG13-iso1 and ALG14 in HEK293 cells using co-immunoprecipitation assays (see Material and methods). HA-tagged ALG13-iso1 was co-expressed with FLAG-tagged ALG14 in HEK293 cells (Table 2). After extraction of the membrane proteins with 1% Triton X-100, HA-ALG13-iso1 was pulled down by anti-HA agarose. FLAG-ALG14 that co-precipitated with the HA-ALG13 was measured by immunoblotted with anti-FLAG antibodies. This experiment demonstrated that FLAG-tagged ALG14 completely failed to coprecipitate with HA-ALG13-iso1, although it efficiently interacted with HA-tagged ALG13-iso2 (Figure 4C). These experiments demonstrated that, unlike the shorter isoform 2, ALG13-iso1 does not display GnTase activity nor form a heterometric complex with ALG14.

To confirm the above results, a yeast complementation assay was performed to test whether ALG13-iso1 has any detectable *in vivo* GnTase activity. HA-ALG13-iso1 and ALG14-FLAG were co-expressed in yeast XGY186 strain (Table 2), in which both *ALG13* and *ALG14* chromosomal loci are deleted. The survival of this strain depends on the galactose-driven promoter *ALG13/14* (*GAL1*pr-ALG13/14), which can be shut off by shifting to growth in medium containing glucose. To determine if ALG13-iso1 and ALG14 have *in vivo* activity, we measured their ability to rescue growth of this yeast strain in the presence of galactose versus glucose. As shown in Figure 4D, HA-ALG13-iso1 and ALG14-FLAG co-expression did not rescue the growth defect caused by the inhibition of yeast Alg13/14; in contrast, HA-ALG13-iso2 and ALG14-FLAG co-expression rescued the growth defect. Consistent with our *in vitro* results (Figure 4B), this complementation assay further confirmed that ALG13-iso1 does not possess GnTase activity *in vivo* or *in vitro*.

### Discriminating the GnTase activity of ALG13- and ALG14-CDG mutants

**FIGURE 5:**
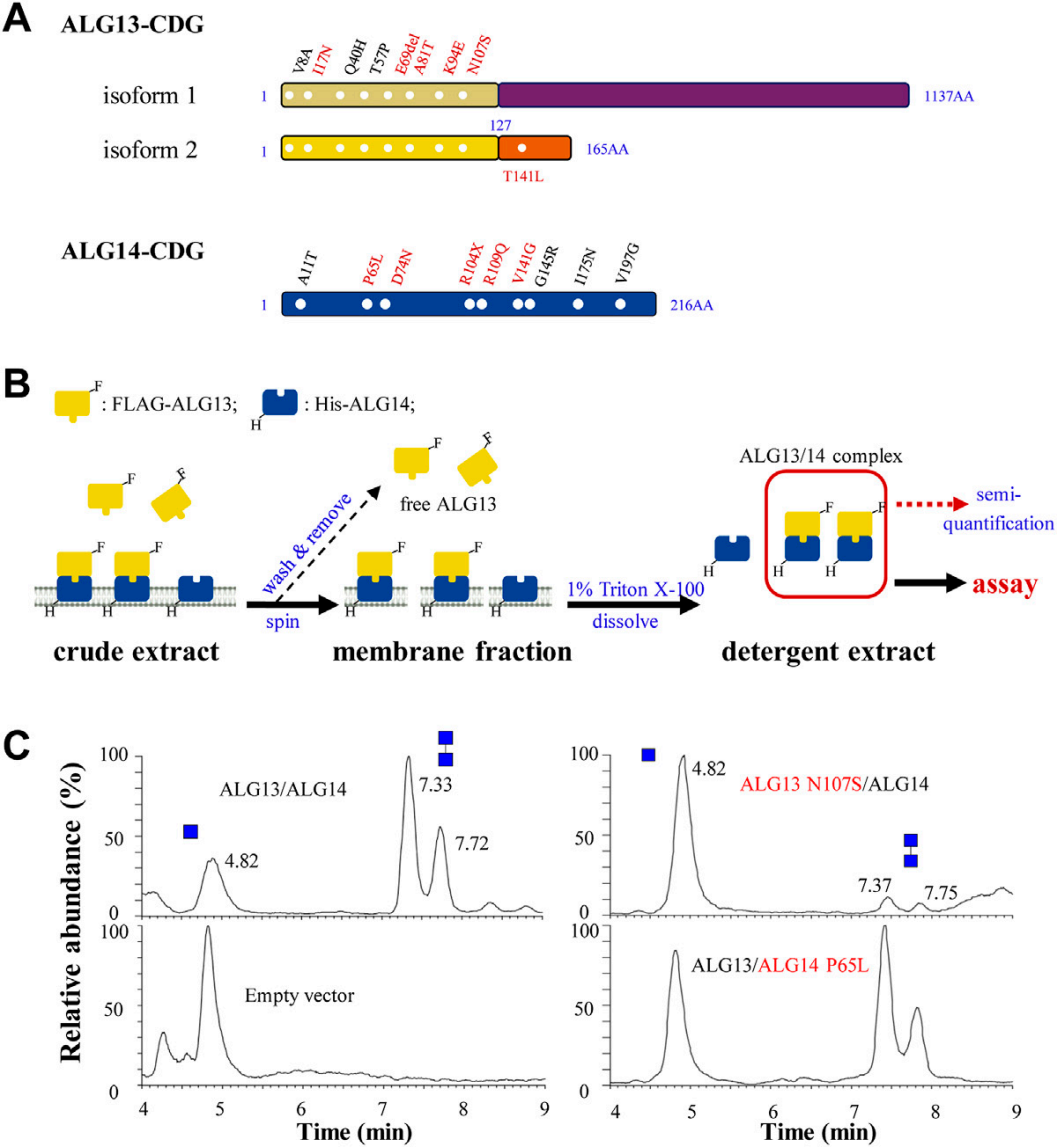
The semi-quantitative assay for ALG13- and ALG14-CDG mutants. **(A)** ALG13- and ALG14-CDG missense mutations distribution diagram. Six mutations in ALG13-iso2 and five mutations in ALG14 (labeled in red) were constructed; **(B)** Schematic diagram of ALG13/14 quantification. *E. coli* cells was broke by sonication and centrifuged to observe the membrane fraction, after washing 3 times by lysis buffer, the membrane fraction was solubilized and the detergent extract was used for GnTase activity assay, and the active ALG13/14 complex amount was semi-quantified using FLAG-ALG13 amount by anti-FLAG antibody; **(C)** The UPLC chromatogram of glycans released from the reaction of wild type ALG13/14, ALG13 N107S/ALG14 and ALG13/ALG14 P65L. Reaction included ALG13/ALG14 (0.1 ng/μl FLAG-ALG13 semi-quantified by western-blot) with the standard reaction mixture for 15 min.

To date, a total of sixteen ALG13-CDG and nine ALG14-CDG missense mutations have been identified from patients (de Ligt et al., 2012; Timal et al., 2012; Esposito et al., 2013; Judith et al., 2013; Smith-Packard et al., 2015; Dimassi et al., 2016; Kobayashi et al., 2016; Bastaki et al., 2017; Hamici et al., 2017; Schorling et al., 2017; Kvarnung et al., 2018; Ng et al., 2020; Alsharhan et al., 2021; Datta et al., 2021; Palombo et al., 2021; Gang et al., 2022; Katata et al., 2022). Among these ALG13-CDG mutations, eight are located in the N-terminal region of ALG13 that is shared by isoform 1 and 2 (Figure 5A), while the remaining seven mutations are located in regions unique to ALG13-iso1, and only one mutation is located in short C-terminal of isoform 2 (data not shown). Based on the results from our study on ALG13 isoforms (Figures 1, 4), we predicted that the eight ALG13-CDG mutations located in the common N-terminal region may affect GnTase activity. To test this idea, we developed a semiquantitative assay to measure the effect of ALG13-CDG and ALG14-CDG mutations on GnTase activity.

We first chose ALG13 N107S and ALG14 P65L as candidates for this analysis as these two mutations have been identified in multiple CDG patients (see Figures 5A,B). These mutations were introduced into plasmids and co-expressed as FLAG (ALG13) or 6His (ALG14) tagged alleles in *E. coli* to produce strains co-expressing wild type (WT) ALG13 + WT ALG14, WT ALG13 + mutant ALG14 P65L or mutant ALG13 N107S and WT ALG14 (see *Materials and Methods*). Membrane fractions containing the ALG13 N107S/ALG14 or ALG13/ALG14 P65L complex were collected and solubilized in 5 ml lysis buffer with 1% Triton X-100 (Figure 5B, see *Material and methods* for details). These detergent extracts were analyzed with anti-FLAG or anti-His antibodies to detect the expression of the ALG13/ALG14 complex (Supplementary Figure S5A). To quickly quantify the concentration of the ALG13/ALG14 complex in the extract, a standard curve method was used, in which purified recombinant yeast Alg13 was used as the standard (Supplementary Figure S5B).

To compare the GnTase activity of the WT and CDG mutants, detergent extracts containing 0.1 ng/μl of ALG13 protein were incubated with the standard reaction mixture at 37°C for 15 min, followed by LC-MS analysis. The use of *E. coli* membrane which is absent from endogenous ALG13/14 would retain the enzyme activity and avoid the laborious protein purification steps. As shown in Figure 5C, WT ALG13/ALG14 complex GnTase catalyzed the production of Gn2-PDol (C95) with a conversion rate of 74%, while the conversion rate catalyzed by ALG13 N107S/ALG14 or ALG13/ALG14 P65L complex was 6% or 59%, respectively. These results demonstrated that ALG13 N107S and ALG14 P65L CDG mutations diminished the GnTase activity of ALG13/ALG14.

Similar to ALG13 N107S and ALG14 P65L, GnTase activities in several other ALG13- and ALG14-CDG mutants (Figure 5A, marked with red) were tested. Plasmids containing FLAG-tagged ALG13-iso2 I17N, E69del, A81T, K94E or T141L were transformed into *E. coli* cells and co-expressed with WT 6His-tagged ALG14. Similarly, plasmids containing 6His-tagged ALG14 R104X, D74N, R109Q or V141G were co-expressed with WT FLAG-tagged ALG13. After confirming protein expression of each ALG13-iso2 and ALG14 variant (Supplementary Figure S5C,E), detergent extracts prepared from each of these *E. coli* strains were assayed for GnTase activity. For a more rigorous comparison with WT ALG13/ALG14 GnTase, the relative activity of each mutation was introduced to show the remaining activity of the ALG13/ALG14 complex mutants. For example, as shown in Table 3, the relative activities were defined using the conversion rate of the mutants/the conversion rate of the WT, and ALG13 N107S/ALG14 and ALG13/ALG14 P65L were calculated as 8% and 80%, respectively. Notably, all of the ALG13/ALG14-CDG mutants that were tested displayed reduced GnTase activity despite the fact that most patients carrying these mutants had a normal N-linked glycosylation phenotype as judged by CDT analysis (Table 2) (Judith et al., 2013; Gadomski et al., 2017; Alsharhan et al., 2021). In conclusion, our results confirmed the GnTase deficiency of these ALG13/ALG14-CDG variants.

**TABLE 3:** CDG mutant primers.

Gene	Primer	Sequence
ALG13	I17N-FW	CCA​CCA​GCT​TTG​ACG​ACC​TCA​ATG​CGT​GTG​TGT
	I17N-RV	ACA​CAC​ACG​CAT​TGA​GGT​CGT​CAA​AGC​TGG​TGG
	A81T-FW	GAT​CTT​GTT​ATT​AGT​CAC​ACA​GGT​GCA​GGA​AGC​TGT​T
	A81T-RV	AAC​AGC​TTC​CTG​CAC​CTG​TGT​GAC​TAA​TAA​CAA​GAT​C
	E69del-FW	GGT​ACA​AGG​ATT​CCT​TGA​AAG​ACA​TTC​AGA​AAG​CAG​ATC​T
	E69del-RV	AGA​TCT​GCT​TTC​TGA​ATG​TCT​TTC​AAG​GAA​TCC​TTG​TAC​C
	K94E-FW	GTT​TGG​AGA​CTC​TGG​AAA​AAG​GAG​AGC​CAC​TCG​TAG
	K94E -RV	CTA​CGA​GTG​GCT​CTC​CTT​TTT​CCA​GAG​TCT​CCA​AAC
	N107S-FW	AGT​GGT​TAT​AAA​CGA​AAA​GTT​GAT​GAA​CAG​TCA​TCA​GCT​GGA​ACT
	N107S-RV	AGT​TCC​AGC​TGA​TGA​CTG​TTC​ATC​AAC​TTT​TCG​TTT​ATA​ACC​ACT
	T141L-FW	GGC​TGT​TAC​AGT​CAA​TGG​ACT​TAT​CAT​TAC​TGA​AAT​GTT​ATC​CTC​C
	T141L-RV	GGA​GGA​TAA​CAT​TTC​AGT​AAT​GAT​AAG​TCC​ATT​GAC​TGT​AAC​AGC​C
ALG14	P65L-FW	CTT​GTC​CAA​TGC​CTA​CTC​ACT​TAG​ACA​TTA​TGT​CAT​TGC​TG
	P65L- RV	CAG​CAA​TGA​CAT​AAT​GTC​TAA​GTG​AGT​AGG​CAT​TGG​ACA​AG
	D74N- FW	GAC​ATT​ATG​TCA​TTG​CTG​ACA​CTA​ATG​AAA​TGA​GTG​CCA​ATA​AAA​TA
	D74N-RV	TAT​TTT​ATT​GGC​ACT​CAT​TTC​ATT​AGT​GTC​AGC​AAT​GAC​ATA​ATG​TC
	R104X-FW	ACC​AAA​TAC​TAC​ATT​CAC​TGA​ATT​CCA​AGA​AGC​CGG​G
	R104X-RV	CCC​GGC​TTC​TTG​GAA​TTC​AGT​GAA​TGT​AGT​ATT​TGG​T
	R109Q-FW	CGA​ATT​CCA​AGA​AGC​CAG​GAG​GTT​CAG​CAG​TCC
	R109Q-RV	GGA​CTG​CTG​AAC​CTC​CTG​GCT​TCT​TGG​AAT​TCG
	V141G-FW	GGT​GAA​GCC​AGA​TTT​GGG​GTT​GTG​TAA​CGG​ACC​AG
	V141G- RV	CTG​GTC​CGT​TAC​ACA​ACC​CCA​AAT​CTG​GCT​TCA​CC

## Discussion

In the second step of the LLO synthetic pathway, the ALG13/14 complex catalyzes the transfer of GlcNAc from UDP-GlcNAc to Gn-PDol, resulting in the formation of Gn2-PDol. This reaction is essential for viability (Gao et al., 2005). Given its conserved role in LLO synthesis, it is reasonable to speculate that ALG13/14 GnTase affects proper N-glycosylation in all eukaryotes. However, contrary to other ALG-CDGs, most reported ALG13- and ALG14-CDG individuals showed essentially normal N-glycosylation (Judith et al., 2013; Schorling et al., 2017; Ng et al., 2020; Alsharhan et al., 2021). This unexpected phenomenon raised doubt about whether or how these ALG13/14-CDG mutations affect the formation of Gn2-PDol in ER LLO synthesis. To verify the causal relationship between ALG13/14 GnTase activity and DEE or CMS phenotypes of their related CDGs, we tried to develop an *in vitro* quantitative assay that directly detects GnTase activity using a recombinant ALG13/14 complex. Our assay system not only enabled us to study the kinetic properties of ALG13/14 GnTase but was also sensitive enough to discriminate the GnTase activities of various ALG13-or 14-CDG mutants.

In previous studies, we successfully used phytanyl-instead of dolichol-linked oligosaccharides as acceptor substrates for enzymatic studies of various ALG GTases (Li et al., 2017a; Li et al., 2017b; Ram Rez et al., 2017; Xu et al., 2018; Li et al., 2019; Xiang et al., 2022). However, when GlcNAc-PP-phytanyl was tested as the acceptor, unlike its yeast Alg13/14, counterpart human ALG13/14 failed to transfer GlcNAc to GlcNAc-PP-phytanyl (data not shown), suggesting a species-dependent substrate specificity of ALG13/14 GnTase. Therefore, in this study, we chose a natural Gn-PDol (C95) that contains 19 isoprene units of lipid tails as the acceptor substrate for the human ALG13/14 GnTase assay. In addition to determining the acceptor substrate, several other useful hints from previous studies were beneficial for development of the *in vitro* GnTase assay. For instance, we knew the formation of the ALG13/14 complex is critical for its GnTase activity (Gao et al., 2008). Moreover, there was no glycosylation modification reported or predicted in databases (https://www.uniprot.org, Q9NP73 and Q96F25; https://www.phosphosite.org) for ALG13 and ALG14. In this study, we tried to purify the active ALG13/14 complex from the *E. coli* membrane fraction by co-expression of ALG13 and ALG14. In contrast, GnTase activity was not detected by simply mixing the individual recombinant ALG13 and ALG14 proteins (Figure 1B). Using the purified ALG13/14 complex, we were able to determine the optimum conditions for the ALG13/14 GnTase reaction and study its kinetic properties (Figure 3). It is noteworthy that unlike other ALG GTases, ALG13/14 GnTase does not require the coordination of metal ions for its activity (Figure 3), demonstrating a metal-independent GTase in the LLO pathway.

We used this *in vitro* GnTase assay to study the splicing variants of human ALG13. Our results demonstrated that the shortest ALG13-iso2 isoform forms the active human ALG13/14 GnTase required for GlcNAc(β1,4)GlcNAc-PP-Dol (Gn2-PDol) production while the long isoform 1, which failed to interact with ALG14 and therefore lacked GnTase activity (Figures 4B,C). This interpretation was supported not only by a direct detection of GnTase activity of HA-tagged ALG13-iso1 immunoprecipitated from the HEK293 cell lysate but also by a complementation test of co-expressed human *ALG13* and *ALG14* in an *ALG13-* and *ALG14*-deficient yeast strain. In these experiments, ALG13-iso1 showed no GnTase activity either alone or in combination with ALG14 (Figures 4B,D), suggesting it cannot form a complex with ALG14, probably due to the lack of the C-terminus α-helical structure. As reported (Gao et al., 2008), the C-terminus α-helix of ALG13-iso2 is required for the interaction with ALG14 to obtain the enzymatic activity, which was supported by the decrease (26%) of a pathogenic mutation in the C-terminus of ALG13 (T141L, Table 2). Bioinformatic and phylogenetic analysis indicated that homologs of ALG13-iso2 are widely conserved among kingdoms of *Fungi*, *Plantae*, *Animalia*, and even in *Protist*, while ALG13-iso1 homologs are only found in *Aves* and *Mammalia* classes of the animal kingdom (data not shown). These findings further support our conclusion that isoform 2 is the only ALG13 isoform required for N-glycosylation. This raises the question of what is the function of ALG13-iso1. Besides containing the N-terminal GnTase catalytic domain, human ALG13-iso1 also contains an ovarian tumor deubiquitinase domain with predicted active sites in its C-terminal region. It has been suggested that ALG13 ovarian tumor deubiquitinase domain may catalyze ubiquitination of certain protein targets, such as GTases in the LLO pathway (Ng et al., 2020). It will be of interest to determine whether or not ALG13-iso1 regulates the activity of one of these GTases, or even ALG13-iso2, via its deubiquitinase activity. Nevertheless, in this study, we confirmed that ALG13-iso1 is not directly involved in ALG13/14 GnTase activity.

The *E. coli* expression system we used does not contain any endogenous interfering GTase activities and therefore has proven very useful for production and analysis of recombinant ALG proteins involved in LLO synthesis (Li et al., 2017a; Li et al., 2017b; Ram Rez et al., 2017; Li et al., 2019). Expression level of ALG13/14 in *E. coli* was approximately 100 folds than that in HEK293 cells. Therefore, we chose *E. coli* expression system to obtain larger amount of ALG13/14 for studying the enzymatic properties and synthesizing N-glycans *in vitro*. This property enabled a convenient semiquantitative GnTase assay, in which the membrane fraction of *E. coli* co-expressing various combinations of human ALG13 and ALG14 alleles were incubated with purified Gn-PDol (C95) acceptor (Figure 5). By omitting the purification step, this assay provided a quick way to test the GnTase activity of ALG13/14-CDG variants. Pathogenic mutants in ALG13 cause an X-linked congenital disorder of glycosylation with variable clinical phenotypes. Due to the lack of a suitable assay system, the effects of ALG13-CDG mutations on N-glycosylation were not yet understood. Using the ALG13 c. 320A>G (p.N107S) mutation as an example, more than 40 patients, including three males with this recurring mutation, were reported to have clear intellectual disabilities. However, all of the tested ALG13 N107S patients presented normal or only subtle deficiencies in N-glycan modifications using isoelectric focusing of transferrin or ESI-QTOF/MS assays (Smith-Packard et al., 2015; Dimassi et al., 2016; Bastaki et al., 2017; Hamici et al., 2017; Ng et al., 2020; Alsharhan et al., 2021). Our semiquantitative assay demonstrated that the ALG13 N107S/ALG14 complex possesses 8% relative activity compared with WT ALG13/14 GnTase (Figure 5; Table 2). The discrepancy between previously published results and those described here demonstrating severe deficiencies of ALG13 N107S emphasizes the importance of having a convenient and sensitive assay for discriminating the N-glycosylation abnormalities in patients with ALG13/14 mutations. Our results confirmed GnTase deficiency as the cause of ALG13/14-CDG. The *in vitro* assay we describe will open new avenues for better understanding N-glycosylation and its relationship to the pathogenicity and clinical severity of CDG.

## Data Availability

The datasets presented in this study can be found in online repositories. The names of the repository/repositories and accession number(s) can be found in the article/Supplementary Material.

## Abbreviations

ALG, Asparagine-linked glycosylation; CDG, congenital disorders of glycosylation; CDT, carbohydrate-deficient transferrin; CMS, congenital myasthenic syndromes; DEE, developmental and epileptic encephalopathy; ER, endoplasmic reticulum; G, glucoses; GTases, glycosyltransferases; Gn, GlcNAc; GnTase, UDP-N-acetylglucosamine transferase; GlcNAc, *N*-acetylglucosamine; Gn-PDol, GlcNAc-PP-Dol; GDP-Man, GDP-Mannose; LC–MS, liquid chromatography/mass spectrometry; LLO, lipid-linked oligosaccharide; M, mannoses; PDol, dolichol pyrophosphate; SDS–PAGE, sodium dodecyl sulfate-polyacrylamide gel electrophoresis; UDP-Glc, UDP-glucose; WT, wild type.
