# Supplementary material for: An in vitro assay for enzymatic studies on human ALG13/14 heterodimeric UDP-N-acetylglucosamine transferase
Source: Front Cell Dev Biol. 2022 Sep 19;10:1008078. doi: 10.3389/fcell.2022.1008078 (PMC9527342; doi:10.3389/fcell.2022.1008078)
Supplement: Supplementary file 1 [file DataSheet1.PDF]

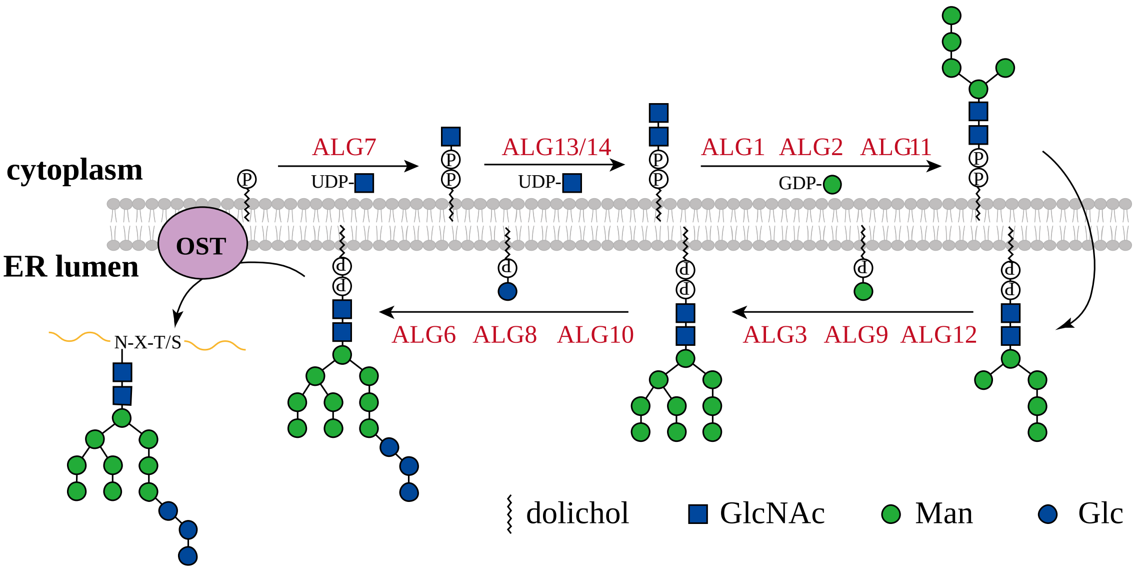

Supplementary Figure 1: lipid-linked oligosaccharide precursor synthesis pathway in ER. The process of lipid-linked oligosaccharide precursor synthesis , fourteen sugars was added by ALG proteins in sequence.

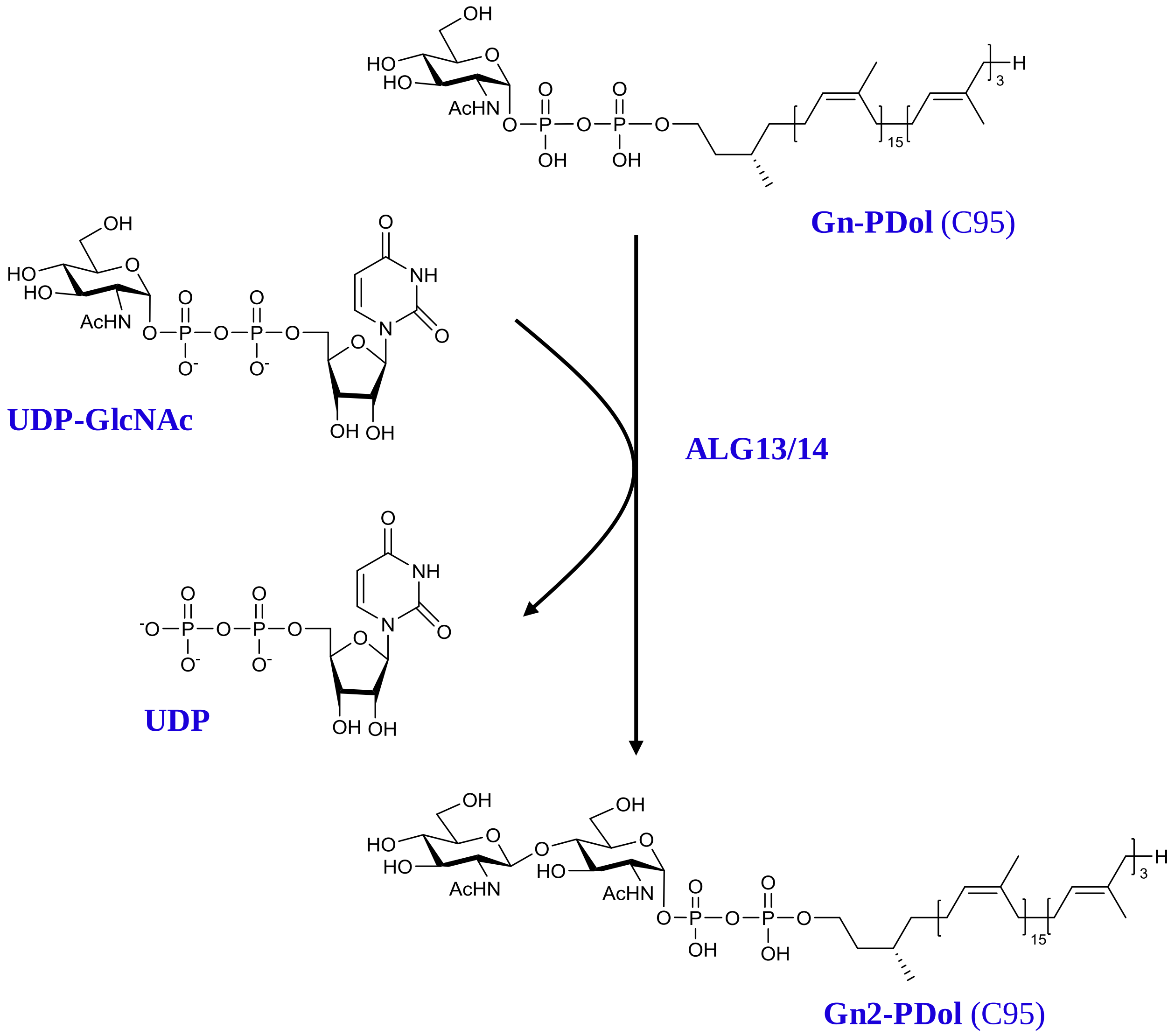

Supplementary Figure 2: Scheme of ALG13/14 GnTase catalyzed reaction.

Percent Identity Matrix

Homo	100.00%	87.27%	59.15%	40.61%	30.67%
Mus	87.27%	100.00%	57.93%	41.82%	30.67%
Danio	59.15%	57.93%	100.00%	45.12%	36.81%
Arabidopsis	40.61%	41.82%	45.12%	100.00%	34.12%
Saccharomyces	30.67%	30.67%	36.81%	34.12%	100.00%

Supplementary Figure 3: Human ALG13-iso2 sequence similarity to animal, plant and fungal cells. Homo: Homo sapiens; Mus: Mus musculus; Danio: Danio rerio; Arabidopsis: Arabidopsis thaliana; Saccharomyces: Saccharomyces cerevisiae;

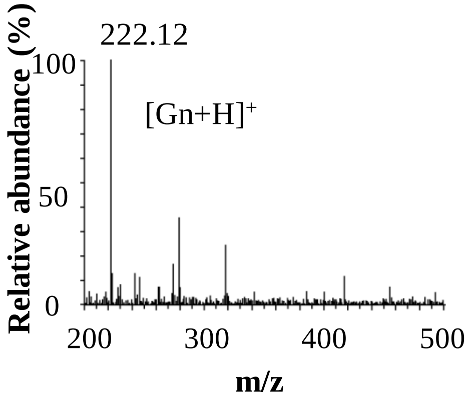

Supplementary Figure 4: Chemical synthesized substrate characterization. ESI-MS spectra of peaks eluted at 4.82 min in UPLC correspond to Gn ($[Gn+H]^+$).

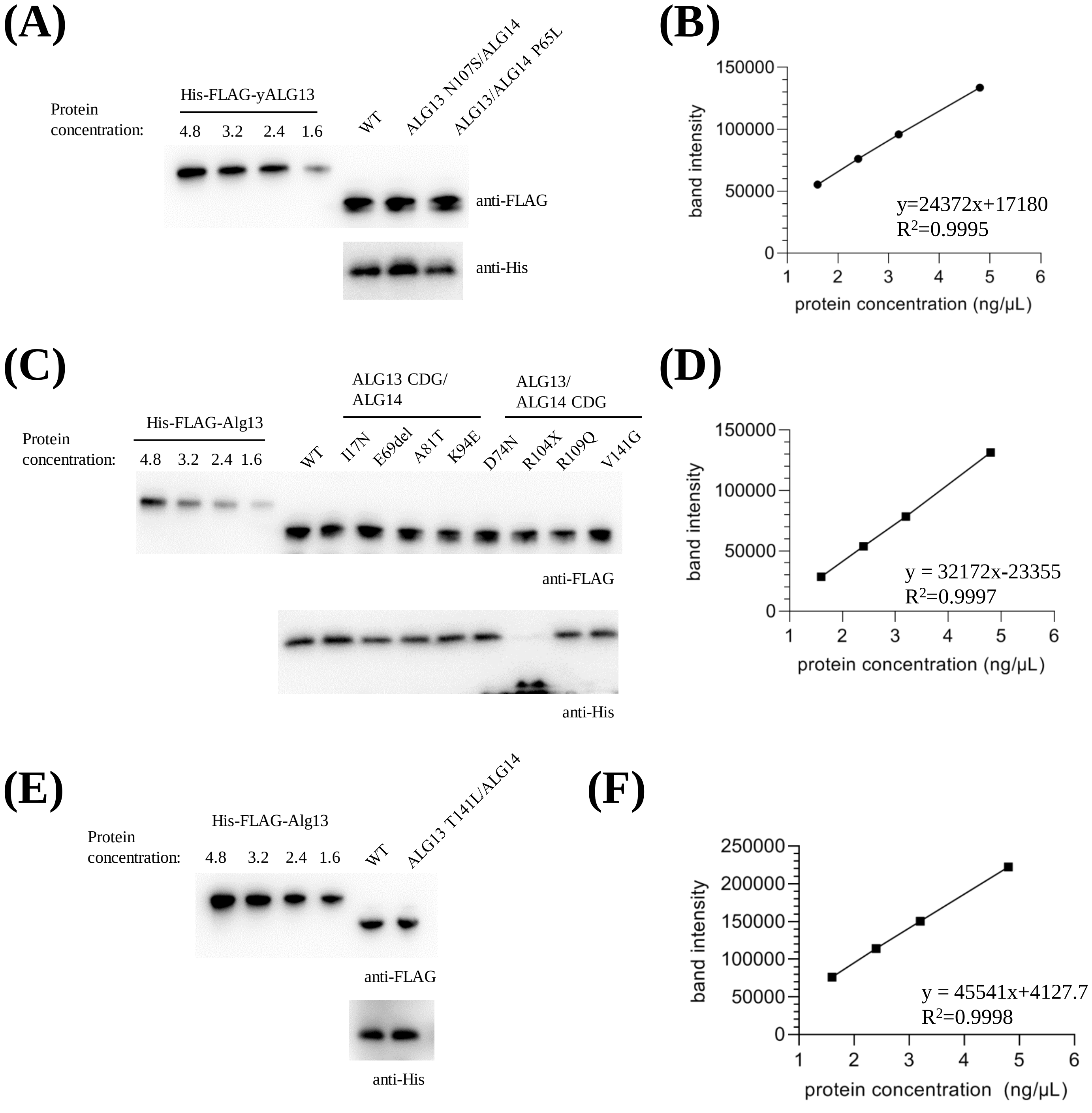

Supplementary Figure 5: Semi-quantification of recombinant ALG13/14 complex. (A) Western blot analysis of recombinant wild type ALG13/ALG14, ALG13 N107S/ALG14 and ALG13/ALG14 P65L expression in *E.coli* detergent extract. Proteins were analyzed using western blot with anti-FLAG and anti-His antibody. Same volume serial dilutions (4.8, 3.2, 2.4 and 1.6 ng/µL His-FLAG-Alg13 protein) purified from *E.coli* were run on the same gels and analyzed with anti-FLAG antibody; (B**)** Semi-quantification of recombinant wild type ALG13/ALG14, ALG13 N107S/ALG14 and ALG13/ALG14 P65L complex in *E.coli* detergent extract. The standard curve was made by the positive correlation between the band intensity and protein concentration. The ALG13 protein concentration was calculated as 6 ng/µL according to the standard curve; (C**)** Western blot analysis of recombinant ALG13/ALG14 and the other CDG mutations expression in *E.coli*; (D**)** Semi-quantification of recombinant ALG13/14 and the other CDG mutation complex in *E.coli* detergent extract as described above. (E**)** Western blot analysis of recombinant ALG13/ALG14 and ALG13 T141L/ALG14 expression in *E.coli*; (F**)** Semi-quantification of recombinant ALG13/ALG14 and ALG13 T141L/ALG14 complex in *E.coli* detergent extract as described above.
